# Supplementary material for: Managing Minds at Work: Development of a Digital Line Manager Training Program
Source: Int J Environ Res Public Health. 2022 Jun 29;19(13):8006. doi: 10.3390/ijerph19138006 (PMC9266047; doi:10.3390/ijerph19138006)
Supplement: Supplementary file 1 [file ijerph-19-08006-s001.zip › Supplementary file S1.pdf]

Supplementary file S1. Checklist of questions to consider when planning, reporting, or analysing co-design activities.

**Checklist item**	**Process / Response**
Aims of the co-design process defined according to user group, specific technology, and context.	Aims provided in stakeholder information sheet aligns with the following definitions:<br>• User group (line managers)<br>• Specific technology (Xerte online package)<br>• Training context (UK employment settings)
Is the target user group identified?	Yes. Line managers are defined as a person with direct managerial responsibility for a particular employee.
At what stage of the research cycle are users involved?	All stages. Initial development (reported here), feasibility and acceptability testing, process evaluation, implementation study.
What are the stages of involvement?	For the development phase (reported here):<br>• Establishing programme theory<br>• Co-development of storyboard content<br>• Review of technical prototype<br>• Usability testing<br>• Approval of final version
What are the underpinning theories?	• *Digital*: Technology Acceptance Model (TAM). Understanding technology acceptance will lead to better prediction of the use of new information resources. Future implementation study will explore confidence in the use of technology, personal control, flexibility, and competent use of information.<br>• *Development*: Collaborative participatory design; user-centred design; Chisholm's Co-design Model and evidence-based (top-down) and experience- based (bottom-up) input<br>• *Participation:* Active learning pedagogy; bidirectional strategies to enable full co-design in eHealth<br>• *Psychological:* 'Universal' mental health promotion [52]
How many users are recruited and how? What efforts are made to ensure they are representative of end-users?	10 expert stakeholders recruited through professional networks and MHPP employers and partners, with experience as line managers across sectors, organisation types and sizes, and/or with lived experience of mental ill-health. Stakeholders included individuals from academic institutions, local authorities, the mental health charity Mind and Public Health England. The stakeholder group had expertise in education and training, specialists in human resources, employment and/or mental health, and line management.
Which aspects of technology design are they involved in?	Programme theory, platform selection, intervention design (module areas, content / storyboard, prototype specification), and implementation (barriers / facilitators)
Are others involved who might provide further insights?	Stakeholders with lived experience of mental ill-health. Experts in digital education and pedagogy.

Which practitioners and researchers are involved?	Stakeholder group includes mental health practitioners. The research team have expertise in occupational and health psychology, mental health, workforce and employment issues, digital health education, and equality, diversity, and inclusion. Digital designers were involved in supporting prototype development.
How are their skills and expertise appropriate to collaborate with end-users, co-develop the technology and understand its context?	Stakeholders and research team have prior expertise in participatory approaches and/or co-design (including digital workforce interventions) and are consortium partners in MHPP. Digital designers and research team have expertise in mental health education and e-pedagogy.
What is the general approach to the activities and facilitation	Agile design, kanban methodology, virtual community of practice.
What methods and techniques are used to engage users?	Multiple methods – Wall Storm (initial ideas proposal), design charette (larger group meeting to sketch storyboard ideas), design jams (smaller group meetings to develop multiple iterations of user experiences), an online data collection form and email communication.
How are these justified in relation to their specific needs and preferences?	Methods and techniques allowed for a highly flexible, user-centred approach to stakeholder engagement (extent of involvement at stakeholder preference). Engagement was virtual due to the increase in remote working during the COVID-19 pandemic.
How does user input influence the prototype development?	Revisions to content (storyboard), design (prototype). Usability testing further refines functionality.
How are activities recorded, documented, analysed, and presented?	Meeting notes, email communications, review summaries, feedback presentations. Findings mapped to appropriate checklists and frameworks.
Are there challenges in the process and how are they approaches?	Conflicting views present challenges. Conflicts resolved through bidirectional strategies to enable full co-design in eHealth: selecting (satisfy one need but not the other), combining (keeping multiple options in the design), integrating (designing a new and coherent functionality that serves both needs) and reframing (redefine perspectives in a way that dissolves the conflict).
Is there an evaluation of the co-design process? What are the methods and findings?	Co-design de-briefs occurred. Qualitative feedback sought from line managers during pilot testing and reported.
Is there an evaluation of the impact of the process on the technology?	Process evaluation will be conducted as part of a randomised feasibility trial which is underway. This will include views towards co-design and relevance of intervention content.
