# Supplementary material for: Managing Minds at Work: Development of a Digital Line Manager Training Program
Source: Int J Environ Res Public Health. 2022 Jun 29;19(13):8006. doi: 10.3390/ijerph19138006 (PMC9266047; doi:10.3390/ijerph19138006)
Supplement: Supplementary file 1 [file ijerph-19-08006-s001.zip › Supplementary file S2.pdf]

Supplementary file S2. Module revisions following stakeholder review

General or Module-Specific	Feedback addressed (Main points)
General	The order of the modules requires revision.<br>'Looking after your own mental health' module is now first as the stakeholders felt this is an essential prerequisite to providing appropriate guidance to employees. Similarly, the cross-module narrative will also be considered after the order and basic architecture is in place.
General	LMs should be encouraged to reflect during each module. Reflective activities were included throughout to help participants see the relevance of the training for their own circumstances, post-module feedback surveys were included and post-training interviews in the subsequent research phase will draw out in-depth insights into the value and implementation of Managing Minds at Work.
General	There should be a degree of flexibility around the timespan for completing the modules to suit individual LMs availabilities. However, an appropriate timescale will be suggested (likely one module per week) and the modules themselves will take no longer than 30 minutes to complete, which the stakeholders thought was reasonable and allowed for LMs workloads/commitments.
General	Reminders should be sent to participants at an agreed schedule which considers individual LM commitments. Logistics for setting up reminders and recommended frequencies are being considered. Flexibility and reassurance should be provided throughout and consider having set times for completion with a calendar invite.
General	When and how to include references to COVID-19 was a divisive issue although it could not be ignored. There is a danger that this will become outdated if the focus is too COVID-19 specific. The suggestion is that this could be navigate by focussing on remote working. However, the impact on essential workers and additional pressures they face should also be considered. Essentially, the stakeholders agreed that COVID-19 needs acknowledging throughout while making sure their learning is generic enough to transfer across all situations.
General	Institutional buy-in can be encouraged by explaining the value of the training, which also needs to be included in introductory text to LMs.
Introduction: Mental Well-being in the workplace	Specific information to include:<br>• How LM[†] can make a difference to MH[‡]<br>• Reference to 'stigma' around MH<br>• Difficulties of remote working (less opportunity for disclosure, spotting warning signs)<br>• One size fits all approach not appropriate<br>• Stress benefits of participating<br>• Other resources are available, and this is about raising awareness

Designing and managing work to promote mental well-being	Consider how to:<br>• Demonstrate best practice through relevant examples<br>• Acknowledge LMs' differing levels of influence<br>• Encourage reflection<br>• Keep to essential information with links to more detailed information<br>• Make a link with other modules
Managing competencies that prevent work-related stress	Specific information to include:<br>• Diverse case-study examples to demonstrate relevance<br>• Details of the support that is available to LMs wishing to make positive changes<br>*This module should come towards the end*
Developing a psychologically safe environment	Further explanation is required:<br>• The role of stigma<br>• How the module builds on the Managing competencies module
Having conversations about mental health at work	This module should:<br>• Build on the foundations of previous modules (introduction and psychologically safe environment)<br>• Offer LMs the opportunity to reflect and build on previous conversations/situations and what they may do differently because of this module<br>• Acknowledge that conversations around MH are not taboo and should be on-going
Looking after your own mental health	Further explanation required:<br>• Looking after their own mental health, reflecting on: What they already do and thinking about what they can improve. Examples they are setting by [not] prioritising their own wellbeing. The pressure they put on their employees<br>Proposal for this module to be earlier / first

†Line Managers; †Mental Health
